# Editorial: Advances in 3D cell culture for drug screening and toxicology evaluation

**DOI:** 10.3389/fbioe.2023.1266506

**Published:** 2023-08-01

**Authors:** Jinglong Tang, Junchao Shi, Jing Liu

**Affiliations:** ^1^ Department of Occupational and Environmental Health, School of Public Health, Qingdao University, Qingdao, China; ^2^ Division of Biomedical Sciences, School of Medicine, University of California, Riverside, Riverside, CA, United States; ^3^ CAS Key Laboratory for Biomedical Effects of Nanomaterials and Nanosafety, National Center for Nanoscience and Techonology of China, Beijing, China

**Keywords:** 3D cell culture, organoid, cell-cell interaction, alternative toxicology, drug screening, toxicology evaluation

Over the past few decades, remarkable innovations in biotechnology and biomaterials have revolutionized drug discovery and toxicology evaluation. Traditional two-dimensional (2D) cell culture methods have served as the cornerstone for chemical screening over the decades. However, their limitations in accurately representing the complex cellular environment have become evident. On the contrary, animal testing has provided valuable evidence for biomedical research, but the high costs and ethic concerns impose restrictions on large scale usage (Breslin and O'Driscoll, 2013). To remedy these limitations of 2D cell culture and animal testing, the development of 3D cell culture technology has emerged as a promising solution to bridge the gap by better mimicking the *in vivo* conditions of different organ systems and offering a more accurate representation of physiological responses to drugs and toxins (Tang et al., 2020). 3D cell cultures provide a more realistic microenvironment for cells to interact and respond to stimuli, including soluble factors, extracellular matrix components, and mechanical forces. Furthermore, they offer a platform for high-throughput screening technologies, accelerating the discovery and development of new drugs and the identification of potential toxins (Li and Belmonte, 2019). Therefore, 3D cell cultures have emerged as a promising alternative to 2D models for drug screening and toxicology evaluation.

The adoption of 3D cell cultures has already demonstrated a significant impact in various fields, including environmental toxicology, tissue engineering, and cancer biology. In this topic, Jiang et al. utilized a 3D human airway organoid (hAO) model to address urgently needed toxicological models for assessing air pollutants. By using primary human bronchial epithelial cells (HBEC), they generated human airway organoids (hAOs) consisting of basal cells, ciliated cells, goblet cells, and club cells. The hAOs exhibited reduced size, significant cell apoptosis, oxidative stress, inflammation response, and an imbalance of cell types after exposure to tire wear microparticles. Fritschen et al. presented a detailed study of seven different bioinks, focusing on a functional liver carcinoma model with 3D-bioprinting technology. Their formulations were characterized for mechanical and rheological properties, as well as albumin diffusivity. They exemplarily demonstrated cellular behavior for HepG2 cells, monitoring viability, proliferation and morphology over 14 days. The observation allowed them to identify the strengths and weaknesses of each material could be identified, resulting in a valuable material portfolio. Wang et al. developed an efficient and reproducible agarose hydrogel microwells to generate uniform-sized multicellular tumor spheroids, offering a better mimicry of conventional solid tumors for precise representation of anticancer drug candidates. By employing high-content screening 3D models, they achieved reliable and high throughput drug cytotoxicity assessments, identified parthenolide as a potential drug that could significantly enhance clinical efficacy for FGFR4 positive hepatocellular carcinoma patients.

Despite the significant advantages of 3D cell cultures over 2D cell cultures, there are several challenges that need to be addressed for their widespread adoption in drug discovery and toxicology evaluation (Kim et al., 2020). The first challenge lies in the standardization of techniques for culturing and maintaining 3D cell cultures. The lack of consensus on the optimal protocols for preparing and maintaining 3D cell cultures, which can lead to result variations among different research groups. Therefore, efforts must be made to develop standardized protocols for 3D cell culture preparation and maintenance. The second challenge is the complexity of 3D cell cultures, making data acquisition and analysis more challenging. The vast amounts of data generated by 3D cell cultures require sophisticated analytical tools to extract meaningful information. However, this challenge can be addressed through the development of computational models and image analysis algorithms. Finally, the cost and time required for obtaining 3D cell cultures can be limiting factors. The specialized equipment and reagents needed for 3D cell cultures can make them expensive to produce, and the time required for obtaining 3D cell cultures can be longer than that for 2D cell cultures. Therefore, efforts must be made to optimize the production of 3D cell cultures and reduce their cost (Sun et al., 2021).

In conclusion, 3D cell cultures hold the potential to revolutionize drug discovery and toxicology evaluation by bridge the study gaps between microscopic cells and macroscopic integrated body with mesoscale methods. They provide a more realistic representation of the physiological responses to drugs and toxins and offer a platform for high-throughput drug screening technologies. However, before their widespread adoption, several challenges must be addressed, including standardization of techniques, development of analytical tools, and optimization of production methods. With these challenges tackled, 3D cell cultures can become a valuable tool for improving the success rate of clinical trials and ensuring the safety of drugs.
